# Antimicrobial Activity of a Novel Cu(NO_3_)_2_-Containing Sol–Gel Surface under Different Testing Conditions

**DOI:** 10.3390/ma14216488

**Published:** 2021-10-28

**Authors:** Daniela Toplitsch, Jürgen Markus Lackner, Alexander Michael Schwan, Andreas Hinterer, Philipp Stögmüller, Kerstin Horn, Natalie Fritzlar, Andreas Pfuch, Clemens Kittinger

**Affiliations:** 1 D&R-Institute of Hygiene, Microbiology and Environmental Medicine, Medical University of Graz, 8010 Graz, Austria; daniela.toplitsch@medunigraz.at; 2 Joanneum Research Forschungsges m.b.H., Institute of Surface Technologies and Photonics, Laser and Plasma Processing, Leobner Str. 94, 8712 Niklasdorf, Austria; juergen.lackner@joanneum.at (J.M.L.); alexander.schwan@joanneum.at (A.M.S.); 3 Inocon Technologie Gesellschaft m.b.H., 4800 Attnang-Puchheim, Austria; a.hinterer@inocon.at (A.H.); p.stoegmueller@inocon.at (P.S.); 4 INNOVENT e.V. Technologieentwicklung, 07745 Jena, Germany; KH1@innovent-jena.de (K.H.); nf@innovent-jena.de (N.F.); ap@innovent-jena.de (A.P.)

**Keywords:** copper, sol–gel surface coating, antibacterial surface, ISO 22196

## Abstract

In this study, assessment of the antimicrobial activity of a novel, plasma-cured 2.5% (*w*/*v*) Cu(NO_3_)_2_-containing sol–gel surface was performed. In contrast to state-of-the-art sol–gel coatings, the plasma curing led to a gradient in cross-linking with the highest values at the top of the coating. As a result, the coating behaved simultaneously hard, scratch-resistant, and tough, the latter due to the more flexible bulk of the coating toward the substrate. Further, the diffusion and permeation through the coating also increased toward the substrate. In our study, tests according to ISO 22196 showed antibacterial activity of the 2.5% (*w*/*v*) Cu(NO_3_)_2_-containing sol–gel surface against all bacterial strains tested, and we expanded the testing further using a “dry” evaluation without an aqueous contact phase, which confirmed the antimicrobial efficacy of the 2.5% (*w*/*v*) Cu(NO_3_)_2_-containing sol–gel surface. However, further investigation under exposure to soiling with the addition of 0.3% albumin, used to simulate organic load, led to a significant impairment in the antibacterial effect under both tested conditions. Furthermore, re-testing of the surface after disinfection with 70% ethanol led to a total loss of antibacterial activity. Our results showed that besides the mere application of an antimicrobial agent to a surface coating, it is also necessary to consider the future use of these surfaces in the experimental phase combining industry and science. Therefore, a number of tests corresponding to the utilization of the surface should be obligative on the basis of this assessment.

## 1. Introduction

Often-touched surfaces are known to act as reservoirs for potential pathogens and, thus, play a distinct role in the transmission of infectious agents [1,2]. Several studies have shown that methicillin-resistant *Staphylococcus aureus* (MRSA) and *Clostridium difficile* (*C. difficile*) spores, and, recently, fungi such as *Candida auris* (*C. auris*) can remain viable on surfaces for several months, posing a huge threat in the healthcare environment [2,3,4,5,6,7]. These findings have prompted the development of a seemingly endless number of no-touch self-disinfecting antimicrobial surfaces and surface coatings since 1964, made from various substances with known biocidal effects, such as copper (Cu), silver, zinc, quaternary ammonium compounds, or the photocatalytic titanium dioxide [8,9]. The antimicrobial properties of Cu were already in use by ancient Egyptians, with recent approaches incorporating different copper alloys in highly touched surfaces such as door handles and bathroom fixtures as a means to prevent nosocomial infections due to the contact-killing mediated via Cu ions released from the surface [2,10,11]. The antibacterial effect occurs via three ways: Cu disrupts bacterial membranes and damages bacterial proteins, as well as induces the generation of highly detrimental hydroxyl radicals via a Fenton-like chemistry, which, in turn, causes subsequent damage to DNA, proteins, and lipids [3]. The mere application of an antimicrobial substance on a surface is usually not enough [10]. In a best-case scenario, anti-microbial-coated surfaces are supposed to offer a variety of functions: long-term effectiveness, and resistance to cleaning agents and cleaning procedures [3]. This combination of properties requires a corrosion-inhibiting and reservoir-forming surface for long-lasting behavior, e.g., a multilayer coating with high adhesion to the chosen substrate. One way this could be realized at low-cost, as well as ease of application, could be sol–gel-based coating systems.

In general, sol–gel synthesis can be used to produce inorganic or organic–inorganic (hybrid polymer) coatings from colloidal dispersions, whereby the chemical process technology first requires a hydrolysis reaction of the alcoholate precursor (formation of “sol particles”), which are then catalytically condensed into chains by acid or base addition [12]. Precursors are often based on silicon (TEOS, TMOS), titanium, and zirconium compounds. By incorporating appropriate organofunctional silicon compounds into the inorganic network during condensation, the properties of the resulting sol–gel layers can be specifically adjusted (e.g., hybrid layers as a combination of hard ceramic and soft/tough polymer properties) [13,14]. Antimicrobial properties can be achieved by incorporation of metal-oxide particles, which are precipitated as nanoparticles by thermal treatment of the layers (>300–500 °C) or are added to the sol–gel as sub-micro particles. Besides Ag, TiO_2_, and Zn, boron (basis for fungicidal boric acid) and chitosan are commonly used additives [15]. Sol–gel coatings are mainly applied by dip-coating and spray-coating on 3D surfaces [16], followed by curing at an elevated temperature and/or photochemically under UV radiation (with the addition of photoinitiators), which decisively influences the coating properties and poses a problem if the coating substrate is a polymer. In turn, using an atmospheric-pressure plasma flow (very short impact of high temperature and intense UV radiation) for the curing not only impacts a lower-temperature load for the substrate and thus allows the use of polymers, but also, in contrast to low-/high-pressure plasma, a cost-intensive reaction vessel (e.g., vacuum chamber) is not necessary. The combination of a functionalized sol–gel coating with curing using atmospheric plasma results in a depletion of carbon at the surface and the formation of a very dense oxide barrier layer, with a gradient into the silicone-polymeric interior, producing a hard and scratch-resistant coating.

Most commonly used for the determination of antimicrobial activity is the international standard ISO 22196 *Measurement of antibacterial activity on plastics and other non-porous surfaces* (also known as Japanese industrial standard (JIS Z 2801)) and the US EPA *Protocol for the Evaluation of Bactericidal Activity of Hard, Non-Porous Copper Containing Surface Products.* ISO 22196 measures the antimicrobial activity of a surface by a reduction in viable bacteria after their incubation on a surface under wet conditions (99% relative humidity (RH), 37 °C). The method is designed to allow optimum contact of antimicrobial substances and bacterial inoculum to promote bactericidal effectiveness, as it was initially designed to evaluate a broad range of antimicrobial products [17,18]. This experimental setup has been criticized by many experts in the field for the artificial experimental conditions used, as a 99% relative humidity and a high temperature of 37 °C do not adequately represent environmental conditions for solid–air interfaces and, thus, the actual activity of the surfaces in a practical setting is not determined adequately [2,4,8,17,19]. The developers of ISO 22196 even recognized the necessity of the further testing of antimicrobial products and proposed a verification test corresponding to the product application.

These varieties of testing methods impede a comparative assessment of the antimicrobial performance of newly developed and/or already established antibacterial surfaces, as testing according to the widely used ISO 22196 does not guarantee significant antimicrobial activity in an actual environmental setting, such as in hospitals or in public transportation [19]. Furthermore, it has been shown that different testing methods can produce varying results in terms of the antimicrobial activity; even interlaboratory testing according to ISO 22916 produced differing results in a recent round robin experiment [17,20]. Factors such as incubation time, bacterial starting concentration, the drying time, the physiological state of the bacteria, as well as nutrient concentration in the inoculum, play a key role in the outcome of ISO 22916 [20,21].

In this study, we investigated a novel, atmospheric-pressure plasma-cured 2.5% (*w*/*v*) Cu(NO_3_)_2_-containing sol–gel surface for its antimicrobial effectiveness. In order to assess the antimicrobial activity of the surfaces, we used ISO 22196 as a standard testing procedure for screening of the different surfaces in combination with testing under “dry” conditions in order to investigate their everyday-life suitability. We further included a “cleaning step” and protein soiling to undertake a more holistic approach for the assessment of the antimicrobial activity of the surfaces.

## 2. Materials and Methods

### 2.1. Sol–Gel Components

First, 5 × 5 cm glass carriers were coated with a sol–gel matrix containing either 2.5% (*w*/*v*) Cu(NO_3_)_2_ additive (presumed antimicrobial samples) or a sol–gel matrix without additives (reference samples) and were provided by INNOVENT e.V. Technologieentwicklung, research group plasma technology, Jena, Germany. Carriers were wiped with isopropanol prior to sol–gel application. For the antimicrobial efficacy, TEOS/MTEOS was used in a molar ratio of 1:3. This sol was catalyzed by using 65% by nitric acid at pH 5, after which 2.5% (*w*/*v*) Cu(NO_3_)_2_ was added. The 2.5% (*w*/*v*) Cu(NO_3_)_2_ was dissolved at room temperature with a magnetic stirrer for 5 min, until the color of the mixture was opaque. The sol–gel was then applied to the glass carriers with 8 cross-shaped spray applications at a 250 mm/s speed of the spray head with a 5 cm spacing, resulting in a ~1 µm film thickness. Curing of the sol–gel samples to create copper-containing silicon oxide thin films was carried out primarily by drying under air for 30 min, and then with patented plasma curing (Inocon Technologie GmbH, Attnang-Puchheim, Austria): InoCoat 3 atmospheric-pressure plasma. This plasma source is based on a dc arc discharge and is driven in a nonpulsed regime. A description of the plasma treatment system including a schematic drawing of the setup—in the special case of a coating procedure—can be found elsewhere [22]. The plasma head was operated with argon, and the applied plasma power was in the range of about 5 W (23 V, 220 A). Due to the thermal impact of the plasma jet, the substrate temperature can quickly reach temperatures up to about 300 °C at the surface [23]. The substrates were moved under the plasma head with a working distance of 5 cm and with a speed of 50 mm/s and were passed twice over the carriers: this created a gradient in the crosslinking in the sol–gel, decreasing from the surface to the interface. For the used specimen size of 50 × 50 mm^2^, a grid spacing of 10 mm, and a traverse speed of 50 mm/s, the curing time was approximately 30 s. As the curing parameter provides for two passes, the final treatment time was 1 min. Samples were covered in aluminum foil before transport to the microbiology laboratory and handled under aseptic conditions before testing.

### 2.2. Release Experiments on Sol–Gel Components

**Figure 1:**
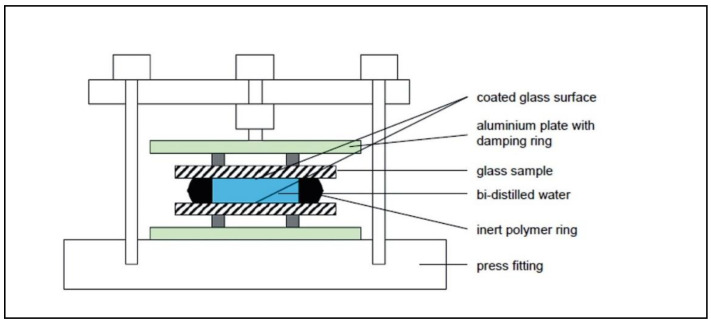
Scheme of the setup for leaching tests.

The release behavior of the sol–gel coatings was investigated by leaching tests (see Figure 1). In this test, distilled water was trapped between 2 glass surfaces coated with Sol Gel + 2.5% Cu and stored at a temperature of 95 °C for 19 h, as described by Palenta et al. [24]. After storage, the water was drawn off (about 5 mL) and analyzed for Na, Mg, Al, Si, K, Ca, Cu, and Sn content by ICP-MS measurement.

Uncoated glass and sol–gel coatings without Cu were tested as references. The leaching behavior was investigated by varying the leaching times.

### 2.3. Scanning Electron Micrographs of the Layers

In order to determine the homogeneity of the layers, scanning electron microscope images (SEM) were taken of the fracture edges of the sprayed sol–gel coating with and without Cu to determine the depth of the holes in the sprayed coating.

### 2.4. Atomic Force Microscopy for Surface Characterization

In order to determine the surface roughness, the films were characterized by atomic force microscopy (AFM) (Asylum Research, MFP 3D-Classic, Wiesbaden, Germany).

### 2.5. Washability Testing

In order to determine film stability, a washability test was conducted according to ASTM D2486. For this, Nylon brushes (Elcometer, Aalen, Germany) and a holder of stainless steel with standardized mass, both conforming to ASTM D2486, and distilled water were used on a washability tester (Simex, Haan, Germany). The number of washing cycles was varied from 10 up to 10,000 cycles. The decrease in the film thickness was measured after 10, 100, 1000, and 10,000 cycles using a profilometer system Alpha Step D600 (Schaefer Technologie GmbH, Langen, Germany).

### 2.6. X-ray Photoelectron Spectroscopy

X-ray photoelectron spectroscopy (XPS) was used for depth profile investigations (Kratos Analytical Ltd., Manchester, UK).

### 2.7. FTIR Measurements

Fourier-transform infrared spectroscopy (FTIR) measurements were realized on plasma-cured MTEOS-TEOS thin films using an interferometer IR MB 3000 (ABB Automation products GmbH, Mannheim, Germany).

### 2.8. Testing of Antibacterial Activity

#### 2.8.1. Modified ISO 22196 (JIS Z 2801)

The antibacterial activity of the surfaces was carried out using a slightly modified ISO 22196 (JIS Z 2801), with the following adaptations: *S. aureus* DSM 346, MRSA DSM 11729, and *E. coli* DSM 1576 (Leibniz Institute DSMZ -German Collection of Microorganisms and Cell Cultures GmbH, Germany) were cultivated overnight on Columbia Blood agar plates (BD, Germany) at 36 °C ± 2 °C. One to two colonies were inoculated in a 1:500 dilution of tryptic soy broth (TSB) (Oxoid, Wesel, Germany) in aqua dest. to reach bacterial solutions with a density of approximately 6 × 10^5^ colony forming units (CFU)/mL using a VITEK^®^ DensiCHEK instrument (Biomerièux, Vienna, Austria). Sterile surfaces were inoculated with 100 μL of the bacterial suspension (due to high hydrophobicity of the tested surfaces) and were then covered by a sterilized 4 × 4 cm PET film (VWR International, Vienna, Austria). The covered surfaces were incubated at 36 °C ± 2 °C with a relative humidity of approx. 99% for 24 h in a wet chamber. For control, bacterial quantification was performed from each tested surface immediately after inoculation. Bacterial quantification was performed by rinsing bacteria off the surface using 10 mL of TSB (Oxoid, Wesel, Germany) containing lecithin (Carl Roth GmbH + Co Kg, Karlsruhe, Germany) and Tween^®^ 80 (Amresco Inc., Solon, Ohio, USA) as disinhibitors, followed by shaking incubation at RT at 220 rpm for three minutes. Appropriate dilutions made with 1x PBS (Carl Roth GmbH + Co Kg, Karlsruhe, Germany) of the rinsing solutions were plated on tryptic soy (TSA) agar plates in duplicate. CFUs were counted after incubation for 24 h at 36 °C ± 2 °C and antibacterial activity was calculated as a reduction in the CFU. Experiments were repeated independently at least two times and the results are shown with a 95% percentile CI, with mean.

#### 2.8.2. Dry Assessment of Antibacterial Activity

Assessment of antimicrobial activity was carried out according to Brühwasser et al. [19], with the following modifications: *S. aureus* DSM 346, MRSA DSM 11729, and *E. coli* DSM 1576 were cultivated on Columbia Blood agar plates (BD, Germany) at 36 °C ± 2 °C overnight. One to two colonies were inoculated in a 1:500 dilution of tryptic soy broth (TSB) (Oxoid, Wesel, Germany) in aqua dest. to reach bacterial solutions with a density of approximately 5 × 10^6^ CFU/mL using a VITEK^®^ DensiCHEK instrument (Biomerièux, Vienna, Austria). Sterile surfaces were inoculated with 10 μL of the bacterial suspension, which was then spread homogenously and dried for four minutes under sterile conditions. For the initial control, bacterial quantification was performed via Replicate Organism Detection And Counting (RODAC) sampling using TSA plates with lecithin and Tween (Merck Millipore, Darmstadt, Germany) as disinhibitors from each tested sample immediately after inoculation. RODAC sampling was further performed after three hours to measure possible antibacterial effects. RODAC (replicate organism detection and counting) sampling using specially designed agar plates that are used for environmental monitoring of surface contamination via pressing the agar plate against the surface to be tested, to transfer bacteria from the surface to the agar plate, was used. Antibacterial activity was determined as a reduction greater than three log CFU from the initial control, as suggested in US EPA protocol *Interim Method for Evaluating the Efficacy of Antimicrobial Surface Coatings* [25]. Experiments were carried out in biological duplicates and repeated two separate times.

#### 2.8.3. Impact of Soiling on Antibacterial Activity

Procedures were performed as described in Section 2.8.1 and Section 2.8.2; however, concentrations of 0.03% and 0.3% bovine albumin (Amresco Inc., Solon, Ohio USA) were added to the 1:500 dilution of tryptic soy broth (TSB) (Oxoid, Wesel, Germany) in aqua dest., and the solution was then used to prepare the bacterial inoculum solution.

#### 2.8.4. Impact of Repeated Use on Antibacterial Activity

Procedures were performed as described in Section 2.8.1 and Section 2.8.2; however, after the first use, samples were wiped using paper towels wetted with 70% ethanol as disinfectant prior to the second use. Experiments were carried out in biological duplicates and repeated two separate times.

### 2.9. Statistical Analyses

Statistical analyses of the experiments were performed using the Mann–Whitney-U test, with GraphPad Prism version 7.04 for Windows, GraphPad Software, La Jolla, CA, USA, www.graphpad.com (accessed on 4 December 2017).

## 3. Results

### 3.1. Characterization of the Coatings

#### 3.1.1. Mechanism of Coating Formation

Hydrolysis reaction occurs while the silica alkoxides react with water under acidic conditions, and species such as Si(OR)_3_OH are formed through the (partial) change of alkoxy to hydroxyl groups. Those species are now able to undergo condensation reactions, either with the alkoxides or with the hydrolyzed forms of TEOS or MTEOS. Copper was incorporated into the coating during the condensation step.

#### 3.1.2. SEM Images of the Coatings

**Figure 2:**
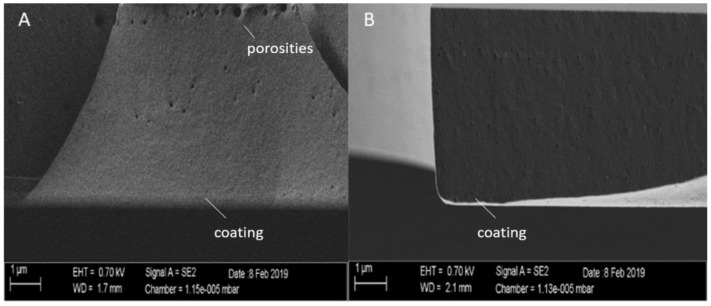
Scanning electron microscope (SEM) images of the layers without (**A**) and with Cu (**B**).

As seen in Figure 2, the fracture edges of the layers shown without (A) and with Cu (B) showed small, enclosed bubbles, which probably came from the spray application of the sol (see Figure 2). The layers were solid and closed, and SEM investigations showed a compact and dense film structure without any visible porosities. There were no visible pores present that passed through the substrate, due to bubble formation. No Cu particles were visible.

#### 3.1.3. AFM Characterization of the Coatings

From the AFM measurements, R_a_ values of about 0.2–0.3 µm were found, with absolute film thickness values of more than 3 µm, independent of whether or not copper was embedded into the film.

#### 3.1.4. Washability Testing

Washability testing revealed a typical film thickness decrease of less than 10% of the original film thickness of about 3 µm, which confirmed good abrasion resistance. It has to be mentioned that certain abrasion failures such as flaking occurred especially at the edges of the used glass substrates.

#### 3.1.5. XPS Depth Profile Investigations

XPS depth profile investigations on copper-enriched sol–gel films showed, in addition to the matrix elements silicon and oxygen and the embedded copper, certain amounts of carbon even in the depth of the films. Moreover, low amounts of nitrogen could be detected all over the sputter depth, which can be explained by an incomplete decomposition of the precursor, resulting in an incorporation of nitrate inside the film matrix [26].

#### 3.1.6. FTIR Measurements

**Figure 3:**
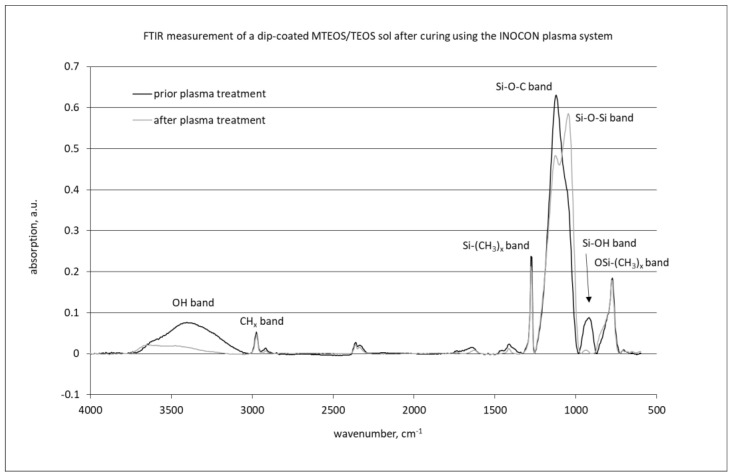
FTIR spectra of as-deposited and plasma-cured MTEOS-TEOS sol–gel film.

As seen in Figure 3, the plasma-cured sol–gel films showed a strong broad absorption between 1000 and 1250 cm^−1^ belonging to Si-O-Si and different Si-O-C vibrations. Strong absorption bands were observed at 2970 cm^−1^, hinting to the presence of (CH_3_), too. Additionally, the band belonging to the Si-(CH_3_)_x_ vibration at 1276 cm^−1^ was pronounced. The region between 800 and 900 cm^−1^ also showed considerable changes. This region is assigned to the Si-OH band (930 cm^−1^) and significant OSi-(CH_3_)_x_-bands between 865 and 750 cm^−1^. Contributions in the spectrum from copper incorporation into the film could not be detected, due to the low agent concentration. After plasma-based curing, we observed a significant intensity decrease of the band at 1250 cm^−1^ belonging to the Si-O-C vibration, as well as a near-disappearance of the Si-OH band at 930 cm^−1^. Additionally, the water content in the film decreased. At the same time, the Si-O-Si band became more pronounced, indicating the formation of the silicon oxide network in the film.

#### 3.1.7. Release Behavior of Sol–Gel Coatings

In all tested samples, there was no flocculation or tinsel observed in the eluates, i.e., there were no detachment or crystallization effects.

The released concentrations of Cu, Na, and Ca remained relatively constant over the elution period. They appeared to reach saturation over the investigated period of 163 h.

**Figure 4:**
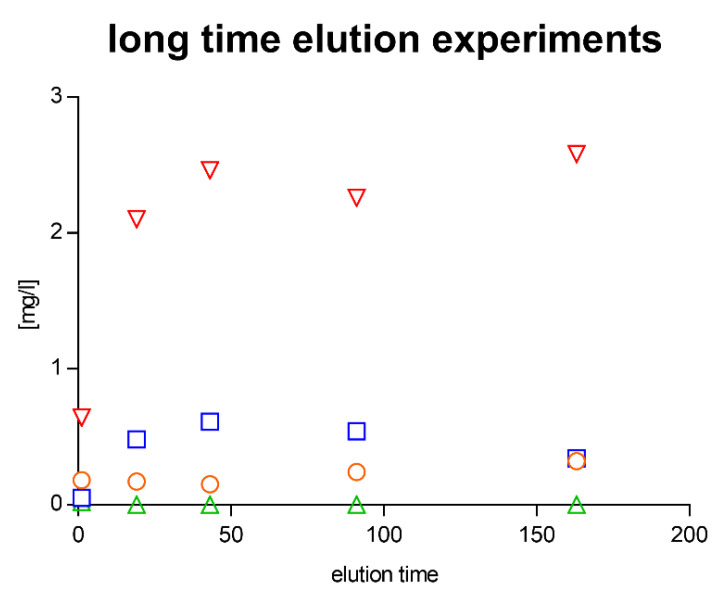
Long-term elution investigations on Cu-doped TEOS/MTEOS-sol–gel layers. Blue squares represent copper, red triangles sodium, orange circles calcium, and green triangles magnesium; elution time is given in hours (values represent 2 independent measurements).

**Figure 5:**
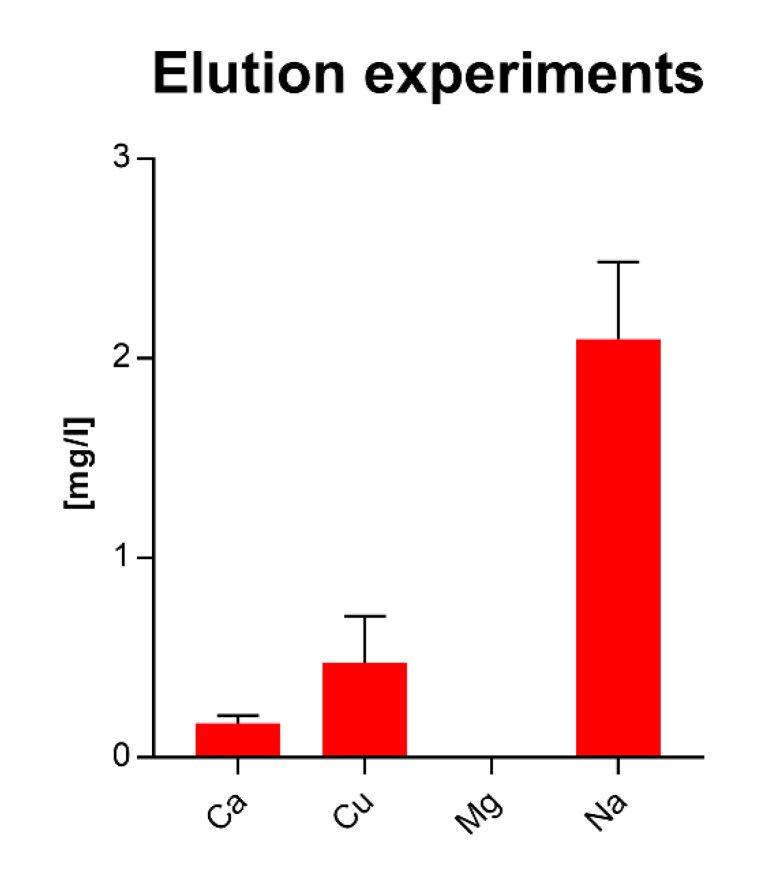
Long-term behavior of Cu release in leaching experiment. The different sol–gel components were after the maximum leaching period of 163 h again exposed to an additional period of 19 h in the same setup. Values for Cu release were low with values between 0.25 and 0.6 mg/L.

The results for the samples indicated that Na was still released from the near-surface areas after the previous 163 h leaching test. Cu and Ca were also released in similar amounts per unit time as during the leaching of the fresh layers (Figure 4 and Figure 5).

#### 3.1.8. Results of Testing According to ISO 22196

**Figure 6:**
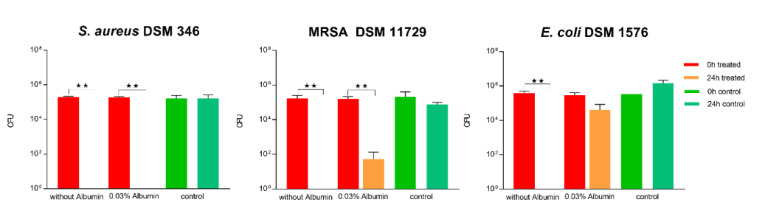
Quantification of viable bacteria using ISO 22196 with low soil load with 0.03% bovine albumin. The results of two independent experiments are shown. A volume of 100 µL of bacterial inoculum was incubated at 99% humidity and 37 °C for 24 h on the 2.5% Cu(NO_3_)_2_-containing sol–gel surface, as well as a sol–gel surface without additive (control). The initial number of bacteria was quantified immediately after inoculation (0 h treated, red; and 0 h control, green) and after 24 h of incubation (24 h treated, orange; and 24 h control, turquoise). Mean numbers of bacteria per surface (CFU) are displayed on a logarithmic scale with error bars indicating the standard deviation of the respective means. Statistically significant differences between the number of viable bacteria after 24 h of incubation are marked. (Mean with 95% CI, Mann–Whitney-U test; ** corresponds to *p* ≤ 0.004).

Test samples were analyzed according to ISO 22196, as described in Section 2.8.1. As demonstrated in Figure 6, the antibacterial activity of the 2.5% Cu(NO_3_)_2_-containing sol–gel surfaces according to ISO 22196 was 5.0 log reduction for *S. aureus* DSM 346 (SA), 6.7 log reduction for *E. coli* DSM 1576 (EC), and 5.0 log reduction for methicillin-resistant *S. aureus* DSM 11729 (MRSA), as, after 24 h of incubation, no viable bacteria remained on the 2.5% Cu(NO_3_)_2_-containing sol–gel surfaces, whereas, in comparison with sol–gel surfaces without any additive, viable bacteria could still be detected after a 24 h incubation period (Figure 6, red bars on the left side).

#### 3.1.9. Impact of Soil Load on Antibacterial Activity

**Figure 7:**
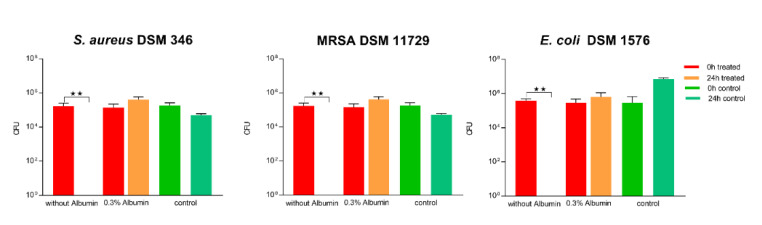
Quantification of viable bacteria using ISO 22196 with high soil load with 0.3% bovine albumin. The results of two independent experiments are shown. A volume of 100 µL of bacterial inoculum was incubated at 99% humidity and 37 °C for 24 h on the 2.5% Cu(NO_3_)_2_-containing sol–gel surface, as well as a sol–gel surface without additive (control). The initial number of bacteria was quantified immediately after inoculation (0 h treated, red; and 0 h control, green) and after 24 h of incubation (24 h treated, orange; and 24 h control, turquoise). Mean numbers of bacteria per surface (CFU) are displayed on a logarithmic scale with error bars indicating the standard deviation of the respective means. Statistically significant differences between the number of viable bacteria after 24 h of incubation are marked. (Mean with 95% CI, Mann–Whitney-U test; ** corresponds to *p* ≤ 0.004).

The surface was then tested according to ISO 22196 in combination with artificial protein soiling (low and high soil load: 0.03% (Figure 6) and 0.3% bovine albumin (Figure 7, respectively). A low soil load of 0.03% bovine albumin did not influence the antibacterial activity of the 2.5% Cu(NO_3_)_2_-containing sol–gel surface against *S. aureus* in ISO 22196, where a 5.6 log reduction could be observed. In contrast to this, 10^2^ log CFU MRSA, corresponding to a 3.2 log reduction, and 10^2^ log CFU *E. coli*, corresponding to a 1.5 log reduction, remained viable on the 2.5% Cu(NO_3_)_2_-containing sol–gel surfaces after 24 h (Figure 6), indicating significantly impaired antibacterial activity of the 2.5% Cu(NO_3_)_2_-containing sol–gel surface. The same experiment was repeated with the addition of a high soil load of 0.3% bovine albumin, which led to the observation of a total loss of antibacterial activity of the 2.5% Cu(NO_3_)_2_-containing sol–gel surface against all three tested strains (*S. aureus* DSM 346, MRSA DSM 11729, and *E. coli* DSM 1576) (Figure 7).

#### 3.1.10. Impact of a Repeated Use on Antibacterial Activity

**Figure 8:**
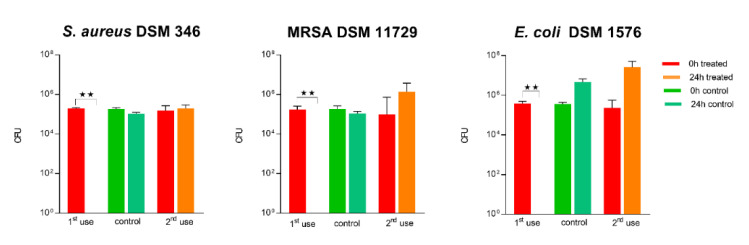
Quantification of viable bacteria using ISO 22196 at first use and at second use. Surfaces were wiped down with paper towels wetted with 70% ethanol. The results of two independent experiments are shown. A volume of 100 µL of bacterial inoculum was incubated at 99% humidity and 37 °C for 24 h on the 2.5% Cu(NO_3_)_2_-containing sol–gel surface, as well as a sol–gel surface without additive (control). The initial number of bacteria was quantified immediately after inoculation and after 24 h of incubation. Mean numbers of bacteria per surface (CFU) are displayed on a logarithmic scale with error bars indicating the standard deviation of the respective means. Statistically significant differences between the number of viable bacteria after 24 h of incubation are marked. (Mean with 95% CI, Mann–Whitney-U test; ** corresponds to *p* ≤ 0.004).

For assessment of the surface durability and stability of the antimicrobial effect, disinfection of previously tested 2.5% Cu(NO_3_)_2_-containing sol–gel surfaces was performed using paper towels wetted with 70% ethanol. As seen in Figure 8, re-use of 2.5% Cu(NO_3_)_2_-containing sol–gel surfaces according to ISO 22196 after disinfection led to a complete loss of antibacterial activity against all three bacterial strains (*S. aureus* DSM 346, MRSA DSM 11729, and *E. coli* DSM 1576) tested.

#### 3.1.11. Results of Testing Using a Dry Assessment

**Figure 9:**
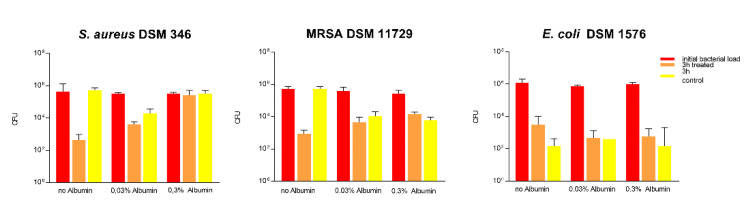
Quantification of viable bacteria using a dry assessment with protein soiling with 0.03% and 0.3% bovine albumin. The results of two independent experiments are shown. A volume of 10 µL of bacterial inoculum (without albumin, with 0.03% albumin, as well as 0.3% albumin) was dried under standard conditions on the 2.5% Cu(NO_3_)_2_-containing sol–gel surface, as well as a sol–gel surface without additive (control). The initial number of bacteria was quantified immediately after inoculation and after 3 h of incubation. Mean numbers of bacteria per surface (CFU) are displayed on a logarithmic scale with error bars indicating the standard deviation of the respective means. The threshold for achieving a mean reduction of three log from the initial bacterial inoculum determines the efficacy of the presumed antibacterial surfaces.

As shown in Figure 9, a dry assessment of the antibacterial activity of the 2.5% Cu(NO_3_)_2_-containing sol–gel surface in comparison with a sol–gel surface without additives was performed for 3 h at room temperature and ~40% RH: the 2.5% Cu(NO_3_)_2_-containing sol–gel surface showed a 5.7 log reduction in viable bacteria for *S. aureus* DSM 346 and MRSA DSM 11729, and a 2.2 log reduction for the *E. coli* DSM 1576 tested. For *E. coli* DSM 1576, on the control surfaces, as well as on the 2.5% Cu(NO_3_)_2_-containing sol–gel surface, a reduction in viable bacteria could be observed, which does not allow a proper comparison of the control with the test object. This may be attributed to the inability of Gram-negative bacteria to withstand desiccation [4].

#### 3.1.12. Impact of Soil on Antibacterial Activity in a Dry Assessment

Assessment of the impact of soiling on the antimicrobial effect of the 2.5% Cu(NO_3_)_2_-containing sol–gel surface was further also tested in the “life-like” dry assessment. The addition of a low soil load of 0.03% bovine albumin did not influence the antibacterial activity against *E. coli,* whereas a 0.7 log reduction could be observed for *S. aureus* and 0.4 log reduction for MRSA. This shows that the antibacterial activity is already significantly impaired when a low soil load is added. The same experiment was repeated with a high soil load of 0.3% bovine albumin, where, again, a loss of antibacterial activity against *S. aureus* and MRSA was observed (as seen in Figure 9). The antibacterial activity against *E. coli* could be again attributed to the inability of Gram-negative bacteria to withstand dry environments, rather than the antibacterial activity of the Cu(NO_3_)_2._

#### 3.1.13. Impact of Repeated Use on Antibacterial Activity in a Dry Assessment

**Figure 10:**
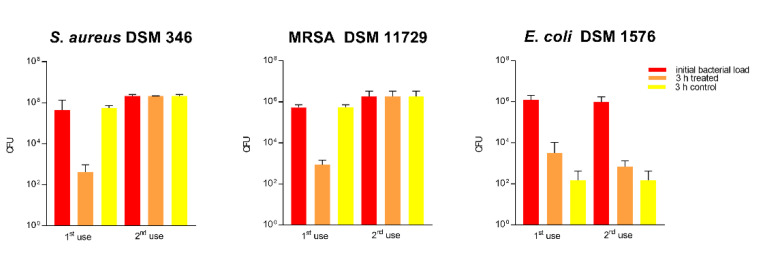
Quantification of viable bacteria using a dry assessment at first use and at second use. The results of two independent experiments are shown. A volume of 10 µL of bacterial inoculum was dried under standard conditions on the 2.5% Cu(NO_3_)_2_-containing sol–gel surface, as well as a sol–gel surface without additive (control), and the experiment was repeated twice after wiping down the surfaces with 70% ethanol. The initial number of bacteria was quantified immediately after inoculation and after 3 h of incubation. Mean numbers of bacteria per surface (CFU) are displayed on a logarithmic scale with error bars indicating the standard deviation of the respective means. The threshold for achieving a mean reduction of three log from the initial bacterial inoculum determines the efficacy of the presumed antibacterial surfaces.

For assessment of the surface durability and stability of the antimicrobial effect of the 2.5% Cu(NO_3_)_2_-containing sol–gel surface, disinfection of already used surfaces was performed using paper towels wetted with 70% ethanol. As seen in Figure 10, the re-use of test surfaces in the “life-like” dry assessment after disinfection led to a loss of antibacterial activity against *S. aureus* and MRSA already at second use (as seen in Figure 9).

## 4. Discussion

A large number of antimicrobial surfaces are being developed or are already on the market, in an effort to reduce infections in high-risk environments such as hospitals and to further aid in minimizing the spread of multi-resistant bacteria such as ESBL or MRSA [27,28,29]. The performance evaluation of such antimicrobial surfaces is mainly performed using the ISO 22196 standard, which is a quick, straightforward assessment technique. However, several researchers have proposed additional testing that verifies the results obtained with the initial ISO 22196 testing. Supplemental methods should correspond to the actual product application, as many studies have shown that an antibacterial effect observed under conditions used in ISO 22196 might not be seen when tested under environmental conditions [4,30].

In our study, initial tests with ISO 22196 (37 °C, 100% RH) showed the antibacterial activity of a 2.5% Cu(NO_3_)_2_-containing sol–gel surface against all three bacterial strains (*S. aureus* DSM 346, MRSA DSM 11729, and *E. coli* DSM 1576) tested, and a further “life-like” dry evaluation (RT, ~40% RH) confirmed these results, which goes in line with the findings in other studies that used pure copper/copper-alloy-based surfaces and demonstrated their antibacterial activity [4,31,32,33]. The antibacterial activity of copper-containing surfaces has mainly been attributed to the damage of cuprous copper ions to Fe-S clusters in proteins needed for general cellular metabolism for aqueous conditions, as well as the damage of the cytoplasmic cell membrane and weakening of the cell wall in dry surroundings [11].

We further broadened our methodological spectrum in order to assess the ability of the surface to provide antibacterial activity even under exposure to soiling and disinfection routines. Organic matter may reduce the activity of the antimicrobially active agent, either by reacting chemically with the disinfectant or by blocking the access of the disinfectant to the bacteria [34]. For this purpose, albumin was used to simulate a low (0.03%) organic load and high (0.3%) organic load, as found in DIN EN 13697:2019 Chemical disinfectants and antiseptics, Quantitative non-porous surface test for the evaluation of bactericidal and/or fungicidal activity of chemical disinfectants used in food, industrial, domestic and institutional areas. In our study, the addition of 0.3% albumin led to a significant impairment of the antibacterial effect according to ISO 22196 and the “life-like” dry evaluation. However, even the addition of only 0.03% albumin impaired the antimicrobial effect of the coating and reduced the effectiveness against *S. aureus* markedly (Figure 6 and Figure 8). This has also been shown by Airey et al. [35], who showed that soiling with 1% albumin reduced the antibacterial effectiveness of polished copper surfaces against *S. aureus*, and that repeated cleaning cycles led to a stronger bond of the bacteria and albumin to the surfaces, which, in turn, increased their resistance against cleaning. Similar results were also obtained by Noyce et al. [36], they observed that soiling with beef juice significantly prolonged the survival of *E. coli* O157 on copper-cast alloys. It is thought that organic compounds such as proteins provide a protective matrix for the bacteria from the copper ions and, in turn, reduce their antibacterial effect [2,3,35,36]. In comparison to this, another study observed an increase in antimicrobial activity when the bacterial inoculum was exposed to albumin, but these results could not be explained yet [37].

We observed that disinfection with 70% ethanol, as it is common practice in hospitals for open hard surfaces [35], led to a total loss of antibacterial activity of the surfaces, which might be explained by mechanical or chemical disintegration of the surfaces themselves (see Figure 8 and Figure 10). This again highlights that testing the durability of these surfaces to withstand cleaning routines needs to be a main consideration for antibacterial surfaces.

## 5. Conclusions

The testing of the antimicrobial activity of surfaces according to the standard method ISO 22196 has a liquid interface. This promotes the mobility of the test microorganisms, so it is easier for them to come into contact with the antibacterial coating, which might contribute to higher antimicrobial reduction rates. Contrary to that, under dry conditions (e.g., 10 µL of inoculum spread over 4 × 4 cm), the test microorganisms are fixed on the surface and so contact with the metal ions could be hindered. Additionally, bacteria suffer from desiccation under dry conditions, Gram-negative bacteria such as *E. coli* even more than Gram-positive bacteria [38], which was also noticed in our results. These circumstances combined may have led to an additional distortion of the results in a dry vs. a wet environment. For high-touch antimicrobial surfaces, regular cleaning and mechanical resistance are basic requirements and future applications of this technology must consider the durability of the antimicrobial effect, which needs to be assured for a sufficient amount of time [10].

## Data Availability

Data is contained within the article.
